# Entomological Monitoring and Evaluation: Diverse Transmission Settings of ICEMR Projects Will Require Local and Regional Malaria Elimination Strategies

**DOI:** 10.4269/ajtmh.15-0009

**Published:** 2015-09-02

**Authors:** Jan E. Conn, Douglas E. Norris, Martin J. Donnelly, Nigel W. Beebe, Thomas R. Burkot, Mamadou B. Coulibaly, Laura Chery, Alex Eapen, John B. Keven, Maxwell Kilama, Ashwani Kumar, Steve W. Lindsay, Marta Moreno, Martha Quinones, Lisa J. Reimer, Tanya L. Russell, David L. Smith, Matthew B. Thomas, Edward D. Walker, Mark L. Wilson, Guiyun Yan

**Affiliations:** The Wadsworth Center, New York State Department of Health, Albany, New York; Department of Biomedical Sciences, School of Public Health, State University of New York, Albany, New York; The W. Harry Feinstone Department of Molecular Microbiology and Immunology, The Johns Hopkins Malaria Research Institute, Johns Hopkins University Bloomberg School of Public Health, Baltimore, Maryland; Department of Vector Biology, Liverpool School of Tropical Medicine, Liverpool, United Kingdom; Malaria Programme, Wellcome Trust Sanger Institute, Hinxton, Cambridge, United Kingdom; The University of Queensland, Brisbane, Australia; Commonwealth Scientific and Industrial Research Organisation (CSIRO), Brisbane, Australia; James Cook University, Cairns, Australia; Malaria Research and Training Centre, Faculty of Medicine Pharmacy and Dentistry, University of Sciences, Techniques and Technologies of Bamako, Bamako, Mali; Department of Chemistry, University of Washington, Seattle, Washington; National Institute of Malaria Research, National Institute of Epidemiology Campus Chennai, Tamil Nadu, India; Papua New Guinea Institute of Medical Research, Madang, Papua New Guinea; Infectious Diseases Research Collaboration, Kampala, Uganda; National Institute of Malaria Research, Field Unit Goa, Goa, India; School of Biological and Biomedical Sciences, Durham University, Durham, United Kingdom; Division of Infectious Diseases, University of California, San Diego School of Medicine, La Jolla, California; George Palade Labs, University of California, San Diego School of Medicine, La Jolla, California; Public Health Department, Faculty of Medicine, National University of Colombia, Bogotá, Colombia; Papua New Guinea Institute of Medical Research, Goroka and Madang, Papua New Guinea; Pacific Malaria Initiative Support Centre, School of Population Health, University of Queensland, Herston, Australia; Australian Centre for Tropical and International Health, University of Queensland, Herston, Australia; Queensland Tropical Health Alliance, Faculty of Medicine, Health and Molecular Sciences, James Cook University, Cairns, Australia; Department of Entomology, Pennsylvania State University, University Park, Pennsylvania; Center for Infectious Disease Dynamics, Pennsylvania State University, University Park, Pennsylvania; Spatial Ecology and Epidemiology Group, Department of Zoology, Oxford University, Oxford, United Kingdom; Fogarty International Center, National Institutes of Health (NIH), Bethesda, Maryland; Sanaria Institute for Global Health and Tropical Medicine, Rockville, Maryland; Department of Entomology, Michigan State University, East Lansing, Michigan; Department of Epidemiology, School of Public Health, University of Michigan, Ann Arbor, Michigan; Program in Public Health, College of Health Sciences, University of California at Irvine, Irvine, California

## Abstract

The unprecedented global efforts for malaria elimination in the past decade have resulted in altered vectorial systems, vector behaviors, and bionomics. These changes combined with increasingly evident heterogeneities in malaria transmission require innovative vector control strategies in addition to the established practices of long-lasting insecticidal nets and indoor residual spraying. Integrated vector management will require focal and tailored vector control to achieve malaria elimination. This switch of emphasis from universal coverage to universal coverage plus additional interventions will be reliant on improved entomological monitoring and evaluation. In 2010, the National Institutes for Allergies and Infectious Diseases (NIAID) established a network of malaria research centers termed ICEMRs (International Centers for Excellence in Malaria Research) expressly to develop this evidence base in diverse malaria endemic settings. In this article, we contrast the differing ecology and transmission settings across the ICEMR study locations. In South America, Africa, and Asia, vector biologists are already dealing with many of the issues of pushing to elimination such as highly focal transmission, proportionate increase in the importance of outdoor and crepuscular biting, vector species complexity, and “sub patent” vector transmission.

## Introduction

The unprecedented global efforts for malaria elimination in the past decade have resulted in the reduction of malaria cases in several settings,^1^ but also in dramatic increases in resistance to pyrethroids and other insecticides,^2^ changes in the relative importance of outdoor (residual) malaria transmission, and major shifts in biting time, for example, *Anopheles farauti* in the Solomon Islands^3^ and *Anopheles funestus* in Benin and Senegal.^4^,^5^ Together these new trends have already resulted in quantifiable changes in human–vector interactions in several endemic areas, and threaten to jeopardize future gains. Long-lasting insecticidal nets (LLINs) and indoor residual spraying (IRS) and have been the mainstays of malaria control and have had a major impact on reducing global malaria, particularly where vectors are primarily endophagic (indoor biting), endophilic (indoor resting), and anthropophilic.^6^

As such, the goal of global malaria elimination will require additional interventions and improvements in both the application of current control measures and entomological monitoring.^7^ The single biggest threat to sustainable malaria control is insecticide resistance, which has reached alarmingly high levels in some vector populations of Africa, India, and China (M. L. Quiñones and others, unpublished data).^2^ Second, there are indications of local adaptation in vector biting behavior, possibly in response to reliance on LLINs and IRS.^3^,^5^,^8^,^9^ Whether this reflects a lack of vector ingress because of physical barriers, that is, mosquito-proof houses, adaptation of endophagic vectors to exophagy (outdoor feeding), or selection on phenotypic plasticity, is unknown.^10^,^11^ It has been hypothesized that in some areas endophagic populations may have been eliminated, leaving the inadequately controlled exophagic population.^12^ Also, in the Solomon Islands during the 1970s malaria eradication campaign, late night biting of *Anopheles koliensis* and *Anopheles punctulatus*, which had been common, virtually disappeared.^13^ Similarly, a switch from endophily to exophily (outdoor resting) has been documented in areas under intense IRS (R. Sloof, unpublished data).^14^,^15^ The third major issue is the recognition that transmission is both focal and heterogeneous and that we urgently need to incorporate, for example, ecological context of mosquito foraging behavior and vector diversity into our transmission models to improve predictive accuracy.^16^,^17^ Fourth, the use of LLINs at high coverage, although extremely effective overall, can alter species composition, which could change transmission patterns and possibly the entomological inoculation rate (EIR) because of different vectorial capacities, biting times, and behaviors, for example, a decrease in *Anopheles gambiae* and a concurrent increase in the relative proportion of *Anopheles arabiensis*.^6^,^18^,^19^

Vectorial systems vary dramatically across regions and countries,^20^–^22^ and this variation will be reflected in how well malaria transmission responds to control. A suggested benchmark for adequate vector control is the decrease and maintenance of EIR below 1, together with epidemiological measures of malaria in humans.^23^ Achieving EIRs of < 1 is especially challenging in those endemic areas where the current approach of IRS and/or LLINS may not be adequate to cover changing transmission scenarios.

**Figure 1.**
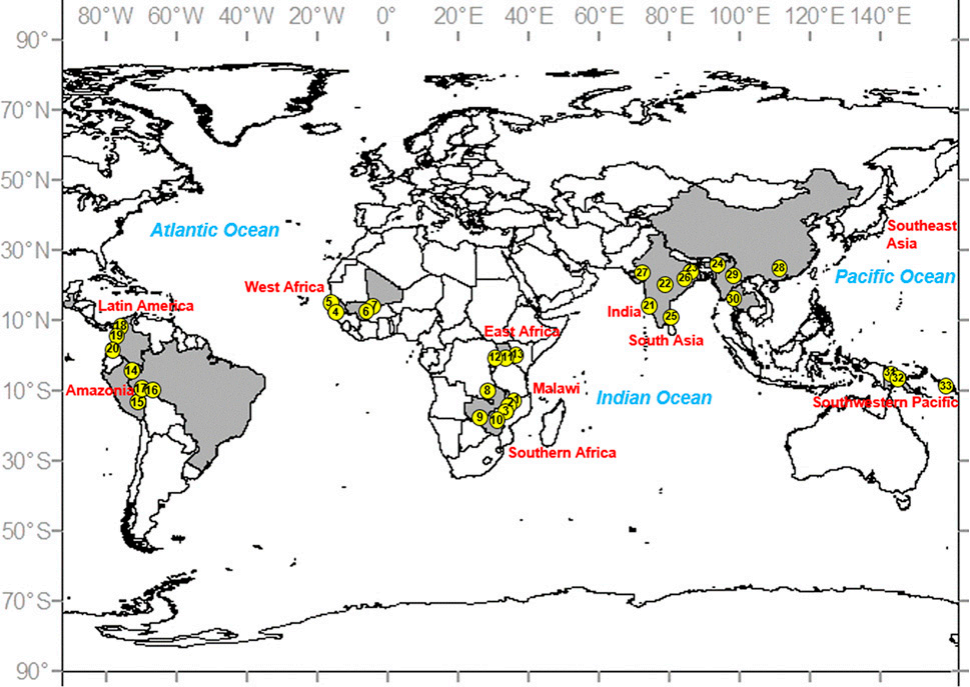
Location of the 33 field sites across 10 International Centers of Excellence in Malaria Research (ICEMR) (in red). The field sites (yellow circles) are numbered consecutively and are described in Table 1.

The United States' National Institutes of Health funded 10 International Centers of Excellence in Malaria Research (ICEMR) in 2010 with a series of common aims including a concerted effort to closely link epidemiology and transmission metrics with vector biology. One of the unique features of the ICEMR program is the focus on longitudinal surveillance sites in diverse epidemiological settings across the globe (Figure 1

). Each ICEMR uses similar approaches and metrics to quantify transmission. The strengths of this approach are our ability to incorporate seasonal and multiyear variation in routine entomological monitoring that can quantify temporal changes in insecticide susceptibility, EIR, vector species composition, and the effects of epidemiology interventions. Such data, when incorporated into malaria transmission models, should increase accuracy and predictive power.

The objective of this article is to provide an introduction to the broad-sense ecology of vectors in the 10 major geographic regions covered by the ICEMR projects and to discuss how similarities and contrasts between the areas will build to a comprehensive view of malaria transmission globally. We are not intending to provide a detailed historical review of transmission ecology in each setting, and as a consequence the extant literature has been sampled broadly but with only limited depth. Vector biologists from each ICEMR selected references that they believed to be the most pertinent to the objective of this article.

## Entomological Metrics at ICEMR Sites

**Figure 2.**
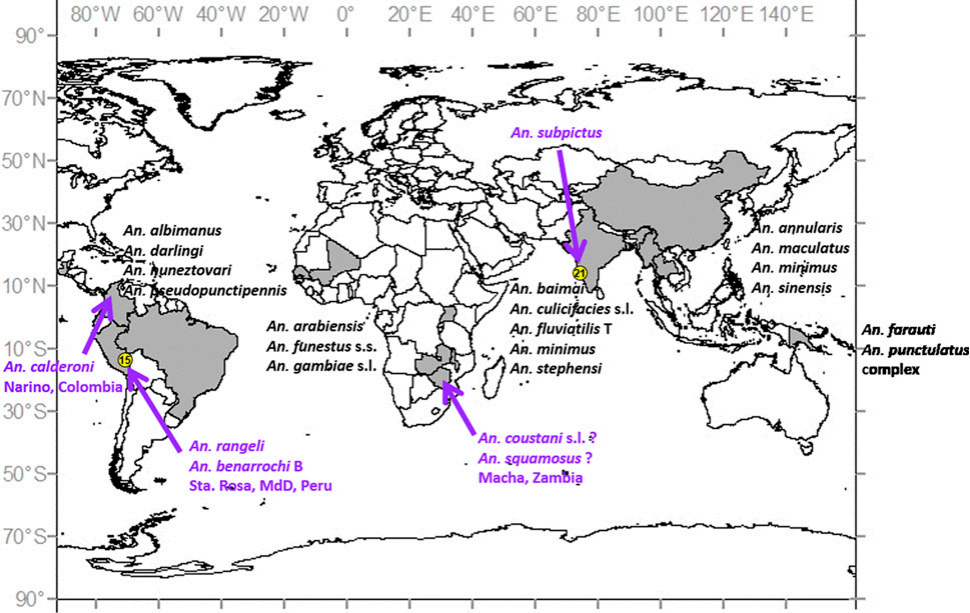
Anopheline malaria vector species across the 10 ICEMRs. New potential vector species, detected in these or affiliated studies, are depicted in purple. In Macha, Zambia, there is not yet conclusive evidence implicating *Anopheles coustani* s.l. or *Anopheles squamosus* as vectors.

Across the ICEMR sites, there is diversity in vector species and their contributions to malaria transmission of, principally, *Plasmodium falciparum* and *Plasmodium vivax* are variable. Although challenging, this is also an extraordinary opportunity to identify commonalities that may lead to new integrated approaches to control and eliminate malaria. Investigations in the 10 major regions are described in brief below, with summary vector biology data (Table 1) together with a corresponding figure of the 33 individual sites geographically located (Figure 1). The wide range of primary vector species, and putative new vectors that several ICEMR studies have detected, is illustrated in Figure 2

.

### Africa.

The four ICEMRs in Africa (Malawi, west Africa, southern Africa, and east Africa; Figure 1) are near exclusively *P. falciparum* transmission settings where malaria is vectored by one or more of the four major African vectors (*An. gambiae* s.s., *Anopheles coluzzii*, *An. arabiensis*, and *An. funestus* s.s.) (Figure 2). Vector control is reliant on LLINs, IRS, or some combination of the two (Table 1). In west and east Africa, vectors are primarily endophagic and endophilic (but see *An. arabiensis*, Table 1), implying that nets should be highly effective, assuming high levels of local coverage. For two of the higher transmission sites in southern Africa, *An. funestus* is similarly expected to be endophagic and endophilic. Although not yet reported for all the African sites, EIRs are, with the exception of the high transmission site in Tororo, Uganda (annual *Pf*EIR ∼125; Table 1),^23^ moderate relative to earlier studies.^1^ A major concern for these ICEMRs is how vectors will respond to the massive rollout of LLINs in sub-Saharan Africa. Already, some populations of *An. gambiae* s.s., such as those in Guinea, have shifted from primarily endophagic biting to primarily exophagic^1^,^8^; whereas in other situations, such as the highlands of Kenya, endophilic *An. gambiae* have been dramatically reduced, with low transmission maintained by *An. arabiensis* and novel anopheline species that are primarily exophilic and bite early in the evening when people are generally unprotected.^12^

### East Africa ICEMR.

Comparing three transmission sites (low, moderate, and extremely high; Table 1) from this Uganda-based ICEMR, only at the low site, Jinja, there was a suggestion of a reduction in *An. gambiae* s.s. and a concurrent increase in the abundance of *An. arabiensis* compared with previous findings.^25^,^26^ The study site in Jinja is peri-urban, and it is possible that the predominance of *An. arabiensis* reflects its adaptation to this disrupted environment as has been observed in west Africa.^27^–^29^

### Malawi ICEMR.

This ICEMR is undertaking studies of *Plasmodium* transmission and malaria risk and prevention in various environments of southern Malawi, particularly in the districts of Blantyre, Chikwawa, and Thyolo (Figure 1).^30^,^31^ Study sites differ considerably, ranging from predominantly rural lowlands in Chikwawa where transmission is intense and essentially year-round, to the rural highlands of Thyolo with moderate seasonal transmission, to sites in and around urban Blantyre City, with apparently lower-level, heterogeneous infection (Table 1). Until the ICEMR-supported research began, little had been published about *Anopheles* vectors in Malawi. One of the first studies by Spiers and others^32^ undertaken in Chikwawa reported that the predominant vectors were *An. arabiensis* and *An. funestus* s.s., although *An. gambiae* s.s. was also present in this Shire River valley area. Other work on filariasis vectors by Merelo-Lobo and others^33^ also found these three species, with *An. funestus* being the most abundant. However, little is known about the relative abundance and role of *Anopheles* species in relation to malaria patterns from Malawi.

Vector ecology and infection studies are now underway as part of two ICEMR-supported projects involving a) health facility-identified, case–control comparisons of urban/peri-urban households in/around Blantyre City and b) cross-sectional, household visit-based sampling across districts of Chikwawa, Thyolo, and Blantyre. In all of these settings, Prokopack-style aspirators and Centers for Disease Control and Prevention (CDC) light traps are being used to test for the presence of indoor adult mosquitoes that are identified by microscopy and confirmed by polymerase chain reaction (PCR) (Table 1). In rural settings, *An. funestus* s.s. is most abundant, with fewer *An. arabiensis* and rare *Anopheles quadriannulatus*. In urban/peri-urban Blantyre, year-round aspiration of 511 households during April 2012 through March 2014 showed that 64% of houses had mosquitoes, with *Culex* spp. representing 98.7% of the sample (M. Wilson, personal communication). Very few *Anopheles* spp. (12 males, 29 females) were found. Nevertheless, more *Anopheles* were captured in households of cases (4.2%) than of controls (1.9%). In this urban setting, it remains very difficult to find many *Anopheles* using aspiration and light traps.

Public health efforts to reduce vector–human contact have used widespread distribution of LLINs and focused use of IRS.^34^ Nationwide distribution of free LLINs has been enhanced since 2012 when ownership was at only 58% of households, and now all children born in health facilities receive an LLIN, as do pregnant women when they first visit an antenatal care clinic. Similarly, a free LLIN is now given to each child at her/his first Expanded Program on Immunization (EPI) visit. From 2008 through 2012, more than 6 million LLINs were distributed in Malawi.^34^ However, *Plasmodium* infection measured by PCR among children under 5 years of age was still 43% overall, and 60.5% in the lowest wealth quintile. The scale-up plan for 2012–2015 aims to achieve one LLIN for every two people in each household.^34^

Other control efforts using IRS are coordinated by the Malawi government, but this program is not nationwide, instead focusing on seven districts of particularly high disease burden: Karonga and Nkhata Bay (northern region), Nkhotakota and Salima (central region), and Mangochi, Chikwawa, and Nsanje (southern region). The 2012 MIS survey indicated that less than 10% of Malawi households had received IRS within the preceding 12 months, suggesting that this form of vector control is relatively less important.^34^

Effectiveness of interventions to reduce vector–human contact depends on where, when, and on whom competent *Anopheles* are feeding, but again little is known about this in Malawi. Recent work in northern Malawi (Karonga) has shown that *An. funestus* and *Anopheles rivulorum* were mostly found indoors, but none were infected with either *P. falciparum* or *P. vivax*.^35^ A new *An. funestus*-like species was also mostly collected indoors, but mainly had fed on animals and also was uninfected. Other studies from countries that border Malawi support the general pattern that both *An. funestus* and *An. arabiensis* predominantly feed indoors and on people. Investigations in southeastern Zambia (∼500 km west of southern Malawi) have shown that *An. funestus* and *An. quadriannulatus* were captured both indoors and outdoors, but nearly all were found to have fed indoors, reinforcing the importance of LLIN use.^36^ More generally, this pattern of *An. gambiae* complex and *An. funestus* group predominantly biting humans indoors at night seems to be common in eastern Africa.^37^

### West African ICEMR.

Three malaria endemic countries: Mali, Senegal, and The Gambia, comprise the focus of this ICEMR (Figure 1). Across this broad geographical area vector populations and malaria transmission differ in their complexity. The site types (riverine: Gambissara, Dangassa; urban: Medina Fall, and rice irrigation: Dioro) were chosen in part to explore differences in length of transmission season, EIR (from ∼5 to 51 bites/month; Table 1), and status of malaria control.^38^ *Anopheles gambiae* and *An. arabiensis* are the major vectors in all the three countries (Figure 2). However, other anopheline vectors are encountered such as *An. funestus* and *Anopheles pharoensis* inland and *Anopheles melas* on the coast.^39^–^43^ In The Gambia and Mali sites, as in the other African ICEMR localities (Figure 1), *An. gambiae* is mainly endophagic, although since the inception of this study, it has been collected feeding outdoors 45–50% of the time (M. Coulibaly, personal communication), similar to the change in behavior documented on Bioko Island, Equatorial Guinea.^8^ *Anopheles arabiensis* is primarily exophagic except in the urban site of Medina Fall where it feeds both indoors and outdoors (Table 1).

Overall malaria transmission is seasonal and coincident with the rainy season. The peaks of transmission occur toward the end of the rains when mosquito densities are waning.^38^ Nevertheless, transmission is perennial in some areas where irrigated rice cultivation maintains anopheline breeding during the dry season.^44^ The urban site of Medina Fall (Senegal) showed the lowest transmission level. The current large-scale vector control strategies in use in Mali, The Gambia, and Senegal are LLINs and IRS. Although the LLINs distribution is country wide at all the three sites through campaigns and routine antenatal consultation and EPI, IRS has been implemented only in targeted areas in the respective countries. None of the west African ICEMR study sites has received IRS to date, but insecticide resistance is widespread.

### Southern Africa ICEMR.

In Macha, Choma District, southern Zambia (Figure 1), there is marked spatial and temporal heterogeneity in the foraging behavior of the main vector *An. arabiensis* and previously undocumented high anthropophily in secondary vectors *Anopheles coustani* s.l. and *Anopheles squamosus* (Figure 2).^45^–^47^ Choma District has the potential to become a malaria-free zone, in part because the formerly primary vector, *An. funestus* s.s., was locally eradicated in 2004, possibly by a drought.^48^ Even though the Macha population of *An. arabiensis* is highly anthropophilic with foraging times that extend from dusk until dawn with a greater tendency for exophagy (Table 1), no specimens have been detected positive for *Plasmodium* since 2006 and transmission in the district remains very low (D. Norris, personal communication).^46^,^49^ Despite the low overall risk, it should be recognized that individual and household risk is very unevenly distributed and spatially clustered, and most importantly, this heterogeneity may be further exacerbated by anti-vector interventions and multiple host feeding by the vector.^16^

In contrast, both endophagic *An. funestus* s.s. and *An. gambiae* s.s. are responsible for very high levels of transmission despite reported coverage of 1.73 LLINs per person (2012) and greater than 90% coverage with IRS (2011) in Nchelenge District, northern Zambia (S. Das, unpublished data).^50^ The inability to achieve control here is likely due to high levels of insecticide resistance to dichlorodiphenyltrichloroethane and deltamethrin and an inability to apply effective control measures to the vector populations that are physically difficult to access or may reside in transient households (D. Norris, unpublished data), a condition further limited by resources.^50^ In Nchelenge District, the two vector species exhibit enormous temporal and spatial heterogeneity, which is hypothesized to exacerbate the observed perennial year-round transmission (S. Das and D. E. Norris, unpublished data) (Table 1). High rates of feeding on multiple human hosts in a single gonotrophic cycle (S. Das and D. E. Norris, unpublished data) and human movement into malaria risk zones are seen as added challenges to control in this area (K. M. Searle and W. J. Moss, unpublished data).

The third site for this ICEMR is Mutasa, eastern Zimbabwe (Figure 1), where resurgent malaria occurs seasonally and *An. funestus* s.s. appears to be the only significant vector. Although current loss of vector control here is likely due largely to insecticide resistance of *An. funestus* s.s. (M. Coetzee, and others, unpublished data), historically *An. gambiae* s.s. was the primary vector in this region.^51^ This change in primary vectors may be attributed to gaps in malaria control because of economic constraints that allowed mainly endophagic *An. funestus* s.s. to invade from nearby Mozambique or emerge from unknown refugia. Insecticides used subsequent to this event, to which the *An. funestus* s.s. population were likely already resistant, would have helped this invasive vector population to fully establish and thrive.

### Latin America.

In marked contrast to the African ICEMR sites, most malaria in Latin America is caused by *P. vivax* (∼70%), except for relatively uncommon hot spots such as Haiti, Guyana, and gold-mining areas across the Amazon, where *P. falciparum* case numbers are higher than the average ∼30%.^1^ In the vast area of the basin drained by the Amazon and its tributaries, *Anopheles darlingi* is the main vector,^52^,^53^ but, as is evident in the Latin American study sites, several other vector species contribute to transmission, and much less is known about their ecologies and entomological metrics (Table 1). Many vector species in the neotropics are exophagic and exophilic (Table 1), with the notable exceptions of *An. darlingi*, *Anopheles albimanus*, and *Anopheles nuneztovari* (see the summary below for Latin American ICEMR), which display endo/exophagy depending on host availability and environmental characteristics.^65^ Therefore, control by IRS has been a mainstay for many years, and, partly for reasons of logistics and distribution, the use of LLINs has spread more slowly in Latin America than Africa, Asia, or the southwest Pacific.^52^,^55^,^56^ An unresolved issue is the relatively high use of IRS combined with very low levels of insecticide resistance (M. Quinones, personal communication).

### Amazonian ICEMR.

There are very few reports in Latin America where *An. darlingi* is no longer the dominant malaria vector, for example, Suriname.^53^ Infrequent, extensive flooding that coincided with the beginning of the interventions in Suriname likely contributed to the local collapse of *An. darlingi*.^53^ *Anopheles darlingi* is the predominant vector in study sites near Iquitos, Peru, and near Acre, western Brazil, in this ICEMR. In these localities *An. darlingi* is the main vector, the most abundant, highly seasonal, exo- and endophagic, and nearly exclusively exophilic (M. Moreno and others, unpublished data) (Table 1).

Despite Ministry of Health and international (e.g., Control de la Malaria en las Zonas Fronterizas de la Región Andina: Un Enfoque Comunitario [PAMAFRO]) efforts to distribute LLINs in Brazil and Peru from 2006 to 2011, unimpregnated net use remains common in some localities, although an integrated approach of LLINs combined with IRS has been recommended.^56^–^59^ Many populations of Amazonian *An. darlingi*, including those in our study sites (M. Moreno and others, unpublished data) display multimodal biting times.^60^ A crepuscular peak (∼19–21 hours) is common, well before most people retire for the evening reducing the potential impact of LLINs.^61^ Major issues in Peru are the correlation of deforestation with significantly high human biting rates along highways^62^ and in riverine settlements (W. Lainhart, unpublished data), and hyperendemic malaria hot spots related to occupational travel.^62^,^63^ In western Amazonian Brazil, deforestation linked to agricultural settlements and gold mining is of primary concern.^56^ In study sites in Madre de Dios region, southern Peru, *An. darlingi* was not common, and both *Anopheles rangeli* and *Anopheles benarrochi* B were detected infected with *P. vivax* for the first time in this region (Table 1, Figure 2), but sample size was so small that the actual role of these species in transmission could not be evaluated (M. Moreno and J. E. Conn, unpublished data). These data suggest that elimination efforts might be concentrated more usefully on the detection and rapid treatment of occupational malaria transmission hot spots. Initial blood meal data (M. Moreno and J. E. Conn, unpublished data) from barrier screens support previous findings from eastern Amazonian Brazil that *An. darlingi* feeds opportunistically, and strongly suggest that host availability is the prime driver of blood meal preference.^64^ It remains to be seen how best to exploit these new findings to improve vector control in this region.

### Latin American ICEMR.

Latin American countries in the Centro Latino Americano de Investigación en Malaria (CLAIM) include Guatemala, Panama, Colombia, and northwestern Peru.^52^ In this broad area, the most important regional vectors are *An. darlingi*, *An. nuneztovari* s.l., and *An. albimanus* (Table 1, Figure 1).^21^ Nevertheless, in one field site in the Pacific region, Tumaco (Figures 1 and 2, Table 1), the species *Anopheles calderoni* was found infected with *P. vivax* (M. L. Quiñones, unpublished data). It was also infected with *P. falciparum* in specimens from a palm-oil plantation in the same region.^55^ These data infer that *An. calderoni* may be relatively important in local transmission, and ecological and biological investigation in addition to control efforts should be increased. There are many critical information gaps for these species, such as lack of data on vector ecology, vector competence, and effects of environmental change on vectors.^54^ The most common malaria control methods have been IRS, LLINs, and early detection, diagnosis, and treatment.^23^ Regrettably, for LLINs, there have been basically no evaluations of the potential suppression of vector populations, vector behavioral changes, transmission level, or location.^54^ Behavioral changes toward increased exo- and endophagy in the northern part of the geographical range of *An. albimanus* (Mexico and Panama) may have been induced by early adoption of IRS.^3^ Overall, the predominant behavior among these species is exophagy and exophily (Table 1); however, in several localities in the Amazon, *An. darlingi* is mainly endophagic and *An. albimanus* displays considerable plasticity, exhibiting both behaviors, depending on host availability and locality.^21^,^65^ Broadly distributed across northern South America, *An. nuneztovari* is more exophagic in the Amazon (where it may be *Anopheles goeldii*) and more endophagic in Colombia and western Venezuela.^66^ As such, decisions on the most appropriate intervention(s) differs across its geographic range necessitating more locally tailored control than that seen in Africa.^65^

New findings of a cross-sectional study using human-landing catch (HLC) in 70 localities in western Colombia (Cordoba, Narino, and Valle), where most transmission occurs, found that *An. albimanus* and *An. nuneztovari* together constituted approximately 80% of the 12,052 adult mosquitoes collected and identified, and these were the only two species positive for *Plasmodium* by enzyme-linked immunosorbent assay (ELISA). Furthermore, 35% of these adults were endophagic. Of all *An. albimanus* collected, ∼22% were endophagic, compared with ∼45% endophagic *An. nuneztovari* (M. L. Quiñones, unpublished data). A survey of breeding sites found that most positive water bodies were either fish ponds or small reservoirs for domestic use; 70% of all larvae were *An. nuneztovari*. At least where *An. nuneztovari* is most abundant and endophagic, continued use of LLINs combined with focal application of larvicides might be the most effective tools, even though malaria elimination in the near term may be difficult to achieve.

### South Asia ICEMR.

Entomological results from this ICEMR have implicated *Anopheles stephensi*, collected during 85 nights from multiple urban and rural localities in Goa, western coastal India, as a vector of *P. falciparum* (Figures 1 and 2). Panaji City, within Goa, had an EIR of 18.1 compared with an overall EIR of 2.35 for all of Goa (Table 1).^67^ In this city, *An. stephensi* is endophagic, but rests outdoors. Most *An. stephensi* (*N* = 55) were actively biting between 03:00 and 06:00, although there were seasonal differences. Both *Anopheles fluviatilis* (*N* = 75) and *Anopheles culicifacies* (*N* = 32) were collected biting humans but neither species was positive for *Plasmodium*. Mosquito control in and around Goa relies on larval suppression using fish and larvicides (Table 1).

In Wardha (central India) and Ranchi (eastern India) (Figure 1), *An. culicifacies* and *An. fluviatilis* are exophagic and exophilic. Here, IRS is the only vector control, and transmission is perennial with peaks during the rainy season. In Ranchi, *An. culicifacies* transmits year long, with peaks during the rainy season, and *An. fluviatilis* transmits primarily during February and March. In Assam state, northeastern India, the vectors are *Anopheles baimai* and *Anopheles minimus*. This is an atypical part of India in which four *Plasmodium* species circulate and are transmitted (Table 1). Malaria is also perennial here. To date, EIRs have not been determined for the localities in Wardha, Ranchi, or Assam.

### India ICEMR.

Urban and rural sites with contrasting transmission dynamics are the main focus of this ICEMR. In urban Chennai, India, a consistent hot and humid climate supports stable, low level transmission of predominantly *P. vivax* malaria by a single vector species, *An. stephensi* (Figure 1, Table 1). Malaria control in Chennai follows strategies adopted by the Urban Malaria Scheme of the national program of India wherein vector control is based on antilarval measures such as the use of abate (temephos), application of *Bacillus thuringiensis israelensis* formulations, and, to a certain extent, larvivorous fish, *Gambusia affinis*. Despite a reduction in malaria prevalence^68^ the disease persists, possibly due to rapid urbanization, regular reintroduction, large numbers of breeding sites, a submicroscopic and/or asymptomatic parasite load, and the difficulty of targeting dormant stages of *P. vivax*. Mosquito control tends to be restricted to application of larval insecticides, targeting known breeding habitats of *An. stephensi* such as wells, overhead tanks, and other water storage containers. Use of interventions against the adult vectors within domestic dwellings, such as LLINs or IRS, is minimal. The reasons for the lack of adult mosquito control are varied but include the extremely dense and complex nature of the housing within urban slum settings (it is logistically challenging to access every house), discomfort in using nets in the hot and humid conditions, low transmission rates (there are many challenges at the household level above and beyond occasional infection with generally nonlethal *P. vivax*), and very low density of adult populations of *An. stephensi*. Indeed, determining where and when local transmission occurs is very difficult. The adult vectors are highly zoophagic (human blood index [HBI] = 0.028) and almost exclusively found in cattle sheds (Table 1). However, these biting and resting behaviors create the potential for novel control strategies targeting the more limited focal sites (i.e., cattle sheds) with tools such as toxic sugar baits, or possibly treating cattle and other livestock directly with insecticides or antihelminthics. Nonetheless, given that the majority of adult malaria vectors are not feeding on humans or resting in domestic dwellings, such focal interventions targeting zoophagic and exophilic behavior could have a dramatic impact on local transmission, which appears to be almost a secondary foraging “spillover” phenomenon.

Vector control practices in forested tribal areas such as Raurkela, follow more established approaches with intensive IRS and LLIN programs. The perennial vector in these rural settings is *An. culicifacies*, which tends to be zoophagic and exophilic. The most common sibling species, B, is refractory to *P. vivax*.^69^,^70^ The primary vector responsible for peak *P. falciparum* transmission is *An. fluviatilis*. This species is restricted to breeding in slow-moving fresh water that occurs post-monsoon, and so exhibits highly seasonal dynamics. The predominant sibling species, S, is an efficient vector, with previous studies showing it to be highly anthropophagic and endophagic, with a HBI up to 0.90.^71^,^72^ Recent results may suggest a shift in *An. fluviatilis* (M. B. Thomas, unpublished data) feeding behavior, mirroring changes observed elsewhere in response to wide-scale use of IRS and LLINs.^8^,^73^,^74^

Studies suggest that *An. fluviatilis* has shifted from resting within human dwellings to semi-enclosed animal sheds.^75^ This apparent behavioral change is actually species replacement; the S type now comprises only 20% of the 2013–2014 population and the zoophagic and exophilic sibling species T, now comprises the majority. This replacement might be due to increased comparative fitness of T during control measures (i.e., some form of competitive replacement), or it could be that S is simply disproportionately affected by interventions with T, feeding in cattle sheds, unaffected by IRS and LLINs, and remaining at similar absolute levels but showing a relative increase. These patterns (including the challenges in interpretation) again mirror those reported elsewhere.^8^,^73^,^74^

### Southeast Asia ICEMR.

The China–Myanmar and Thai–Myanmar border regions have been the geographical emphasis of this project (Figure 1). The Government of China has set a goal of malaria elimination by 2020, and Thailand is pursuing spatially progressive elimination and has a national goal to eliminate malaria from 80% of the country by 2020.^76^ However, high malaria incidence in neighboring Myanmar and cross-border human movement present major challenges for malaria elimination in China and Thailand.^1^ Therefore, understanding vectorial systems and developing site-appropriate transmission control methods in the border regions are crucial.

The study sites on either side of the China–Myanmar border area are separated by less than 10 km but have significantly different vectorial systems. In the Chinese sites (around Nabang town in Yingjiang County, Yunnan Province), the major malaria vectors in the 2010–2012 survey by CDC light traps were *An. minimus*, *Anopheles sinensis*, and *Anopheles maculatus* (Figure 2). *Anopheles minimus* and *An. maculatus* are endophagic and anthropophilic whereas *An. sinensis* is generally exophagic (Table 1).^77^ Anopheline density was highly variable among sites and between seasons. Peak mosquito density was in May, during the rainy malaria transmission season. Molecular taxonomy of a subsample of *An. minimus* s.l. found that the only member of this complex present was *An. minimus* s.s. Moreover, of the anophelines examined, only *An. minimus* was detected infected with *P. vivax*, albeit only one positive individual out of 1,500 tested (0.07%). Blood meal analysis of a modest sample size (*N* = 104) revealed that humans were the main host (82.6%), followed by cattle, pigs, and dogs.^77^ Mixed blood meals of human/pig and human/cow were detected at low frequency (0.9% each). In the Myanmar study sites near Laza Town, Kachin Special Zone, CDC light trap collections in 2013–2014 found *An. minimus* was predominant, constituting 89% of anophelines collected despite high anopheline species diversity (20 species collected). The sporozoite rate of *An. minimus* (1.8%) in Laza Town, Myanmar, was considerably higher than in Nabang Town on the Chinese side.

It is important to note that malaria vector species composition varied significantly by mosquito collection methods. For example, larval mosquito collection in the same study sites in Myanmar found that the four most abundant vectors were *An. sinensis*, *Anopheles barbirostris*, *An. minimus*, and *Anopheles splendidus* (G. Yan, unpublished data) rather than *An. minimus*. Larval and adult survivorship in life table studies found differential survival because of local climate conditions as well as land use and land cover variation (G. Yan, unpublished data). In any predictive model for malaria, the environmental variables that effect anopheline survivorship need to be measured and incorporated.^17^ On the Thai–Myanmar border area, the main vectors are *An. minimus*, *An. maculatus*, and *Anopheles annularis*. All three species rest indoors and outdoors, so LLINs should exert some control on the former portion of their populations.

### Southwest Pacific ICEMR.

The geographic foci of the southwest Pacific ICEMR are the Madang and East Sepik Provinces in Papua New Guinea as well as Central and Western Provinces in the Solomon Islands. Current vector control in Papua New Guinea and the Solomon Islands relies on LLINs with limited use of IRS in the Solomon Islands.^78^ The main malaria vectors across the region are members of the *An. punctulatus* group: *An. punctulatus* s.s., *An. koliensis*, and the *An. farauti* complex, which consists of eight cryptic species (Figure 1), and the diversity of these species varies across sites.^79^–^81^ Two of the primary vectors in the southwest Pacific, *An. koliensis* and *An. punctulatus*, which were endophagic and more anthropophic than *An. farauti*, were dramatically reduced by IRS, and *An. koliensis* may have disappeared from the Solomon Islands.^13^ In Papua New Guinea, two other species in the *An. farauti* group are important vectors: *Anopheles hinesorum* and *An. farauti* 4, with *An. farauti* 6 and *An. farauti* 8 considered to be minor vectors with circumsporozoite antigen detected by ELISA.^82^ Other minor malaria vectors in Papua New Guinea are, either uncommon or with a limited geographical distribution, *Anopheles longirostris*, *Anopheles bancroftii*, *Anopheles subpictus*, and *Anopheles karwari*.^81^,^82^ Collections made by HLC in Papua New Guinea found *An. farauti* s.s. was the predominant species in Mirap village whereas *An. punctulatus* was the most common species found in the inland villages of Yauatong and Wasab in the East Sepik Province. The minor vector, *An. longirostris*, had a relatively high population density in Wasab village. In the Solomon Islands, the only species caught biting humans at significant densities in Central Province was *An. farauti* s.s.

In Papua New Guinea, the populations of *An. punctulatus* and *An. farauti* were mostly zoophilic, late night biting, and exophilic but exhibited both exophagic and endophagic biting habits.^83^ Recent data from the Central Province of the Solomon Islands reported *An. farauti* as highly anthropophagic with more than 90% of blood meals on humans (Table 1).^84^ Both the timing of night biting behavior (from late to early) and location (from indoors/outdoors to predominantly outdoors) of *An. farauti* in the Solomon Islands shifted in response to selective pressure to avoid insecticides following IRS decades ago and these behavioral changes have persisted.^3^ Physiological resistance to insecticides has not been found yet in Papua New Guinea or the Solomon Islands, with the temporal biting shift to earlier possibly providing a behavioral resistance mechanism to minimize exposure of this vector to insecticides.^85^ This change in blood-feeding behavior has appeared independently on multiple islands in this archipelago, suggesting that LLINS and IRS will have a limited impact on malaria transmission for this important regional vector.^86^ Elimination may require the use of supplemental and complementary interventions to be implemented with LLINs.

## Lessons Learned from Vector Biology Across ICEMRS

### Collection methods.

The wide range of trap types and/or control methods favored by each ICEMR is a result of project-specific research questions, trap type collector bias, and controversy surrounding the HLC method because of a perceived infectivity risk to collectors (see Gimnig and others^87^). Furthermore, HLC is expensive and labor intensive. In several of the ICEMR sites, CDC traps combined with either pyrethroid spray catch or aspiration have replaced or supplemented HLC (Table 1). A recent study in the three east Africa ICEMR sites revealed statistically comparable EIRs for CDC compared with HLC such that the former can safely and effectively replace the latter.^25^ On the other hand, the Amazonian ICEMR found that HLC resulted in significantly higher numbers of *An. darlingi* compared with Shannon traps and CDC traps, although the number of infected mosquitoes was so low that EIRs could not be compared (M. Moreno and others, unpublished data).

An important outcome of the ICEMR vector biology studies is a new push to standardize monitoring across sites by the use of barrier screens, recently developed in Indonesia and the south Pacific.^84^ Advantages of this method include simplicity of construction and use of local material, collection of high numbers of both nulliparous and parous anophelines, collection of infected anophelines (to date only evidence from Iquitos; M. Moreno and others, unpublished data), no bias related to feeding preference (humans and animals are not involved as attractants), and physical integrity of specimens collected. The Amazonian, Latin American, southeast Asia, southern Africa, and southwest Pacific ICEMRs are currently testing these traps for effectiveness in collecting resting and host-seeking anophelines across the diverse settings, habitat types, and vector species.

### New putative malaria vectors.

Using standard incrimination criteria (presence of infected, correctly identified anthropophilic vectors concurrent with malaria transmission), evidence of new or potential vector species has been collected in four of the 10 ICEMRs (Figure 2). Although EIRs have been determined only for *An. calderoni* in Colombia, they are in progress for *An. rangeli* and *An. benarrochi* B in southern Peru, and will be calculated, pending vector confirmation, for the additional species and sites (i.e., *An. subpictus* in Goa, possibly *An. coustani* s.l. and *An. squamosus* in Macha).^45^–^47^ In Western Province, Solomon Islands, *Anopheles lungae* is the most common anopheline collected by HLC in several villages. However, incrimination as a potential vector of human malaria awaits confirmation of the presence of sporozoites by PCR or ELISA.^81^,^82^ How these and other putative new vectors will respond to changes in LLIN use or climate change is being actively addressed in the Amazonian ICEMR.^88^

### Value of longitudinal surveillance sites to malaria elimination.

Highlights of the value of these sites thus far include the following:

1. No detectable or very reduced transmission in Choma and Macha, Zambia^46^,^49^; Villa de Buen Pastor, near Iquitos, Peru; Madre de Dios, Peru; and Granada and Remansinho,^24^ western Brazil.
2. Change in the proportion of endophagic to exophagic *An. gambiae* in The Gambia and Mali sites.
3. Species replacement of *An. gambiae* s.s. by *An. funestus* s.s. in Mutasa, Zimbabwe and species replacement of *An. fluviatilis* S by *An. fluviatilis* T in Raurkela, India.
4. Temporal biting shift in *An. farauti* from multiple islands in southwest Pacific archipelago.^85^,^86^
5. Several new putative vector species (see above); evidence for the role of *An. stephensi* in transmission in Goa, India (first EIRs), and *An. minimus* in Yingjiang, China, and Laza, Myanmar; *An. hinesorum*, *An. farauti* 4, *An. farauti* 6, and *An. farauti* 8 in Papua New Guinea.^82^
6. Confirmation of the effectiveness of the barrier trap from studies in the southwest Pacific to collect unbiased samples of outdoor resting mosquitoes (see above).^84^
7. Significant contribution to malaria transmission by *An. albimanus* and *An. nuneztovari* s.s. in 70 localities in western Colombia.
8. Evidence from blood meal analysis near Iquitos, Peru, that *An. darlingi* is more locally opportunistic than anthropophilic.

Each of these discoveries contributes to more accurate EIR values and provides feedback to parallel epidemiological and parasitological studies ongoing in the ICEMR sites.

### Management of outdoor (residual) transmission.

Overall, malaria vector control in the ICEMR study sites is reliant on the two insecticide-based interventions for which there exists a strong, primarily Africa-derived evidence-base: LLINs and IRS.^89^,^90^ However, the ICEMR sites reveal a variety of transmission scenarios that will require a more tailored approach that can be monitored and modified rapidly as the need arises. Where endophagy remains dominant and vectors are resistant to pyrethroids, the deployment of attractive toxic sugar baits (ATSB) indoors in combination with LLINs, is one possibility^91^,^92^ although indoor ATSBs may be temporally unsustainable (S. Lindsay, personal communication). Reduction of crepuscular human–vector contact outside houses might be accomplished by the use of ATSB outdoors, as this intervention was predicted to be especially effective against *An. arabiensis*, which is primarily exophilic.^91^ Furthermore, if these exophilic populations are also mainly zoophilic, treating nearby animal hosts as suggested for *An. fluviatilis*, in India, as mentioned above, could be an effective part of an integrated vector control plan.^93^,^94^ Additional important options to prevent outdoor transmission in the context of integrated control include larviciding, as used in the ICEMR urban sites of Tumaco, Goa, Chennai, and Nadiad (Table 1), and environmental management.^95^,^96^ More broadly, new interventions could include transgenic mosquitoes, sterile male releases, or cost-effective consumer products.

## Conclusions

Data from the ICEMRs clearly illustrate that malaria transmission and vectors are highly spatially and temporally heterogeneous. In addition, behaviors exhibited by many vector species involved are diverse, and although they can be broadly categorized as endophagic, exophagic, endophilic, and exophilic, most vectors exhibit a mix of behaviors (e.g., some “outdoor feeding” vectors will occasionally blood feed indoors). Local behavioral adaptations will require new combinations of sampling, surveillance, and control tools. For example, at one location a program may have to address the control of multiple species, but also the control of a single species that can present multiple behaviors.

## Figures and Tables

**Table 1:** Vector biology and ecology data from ICEMR field sites

ICEMR regional center	Specific site	*Plasmodium* spp.	Major vector(s)	Trap type	Primary behaviors (exo/endo/phagic/philic)	Main control method	Transmission seasonality	EIR (per year)
Malawi	Blantyre District (urban)	*Pf*	*Anopheles funestus* s.s., *Anopheles arabiensis*	CDC, ASP	Endophagic, endophilic (indirect evidence)	LLIN	Rainy season (November–April), low transmission rest of year	N/A
	Thyolo District (rural highland)	*Pf*	*An. funestus* s.s., *An. arabiensis*	CDC, ASP	Endophagic, endophilic (indirect evidence)	LLIN	Rainy season (November–April), low transmission rest of year	N/A
	Chikwawa District (rural lowland)	*Pf*	*An. funestus* s.s., *An. arabiensis*, *Anopheles gambiae* s.s.	CDC, ASP	Endophagic, endophilic (indirect evidence)	LLIN	Perennial, one annual rainy season	N/A
West Africa	The Gambia	*Pf*	*An. arabiensis*, *An. gambiae*	HLC, CDC	Primarily endophagic and endophilic	LLIN, IRS	Rainy season (August–November)	Peak of 23/month
	Gambissara (Upper River)							
	Senegal	*Pf*	*An. arabiensis*	HLC	Primarily exo/ endophagic and endophilic	LLIN	Rainy season peak (August–December)	Peak of 5/month
	Medina Fall (Thiès)							
	Mali	*Pf*	*An. arabiensis*, *An. gambiae*	HLC	Primarily endophagic and endophilic; more recently comparable frequency indoors and outdoors	LLIN	Rainy season peak (July–December)	Peak of 51/month
	Dangassa							
	Koila Bamana (Dioro)	*Pf*	*An. arabiensis*, *An. gambiae*	HLC	Same as Dangassa	LLIN	Virtually perennial; rainy season peak plus irrigation (August–May)	Rainy season peak = 5/month
South Africa	Zambia	*Pf*	*An. funestus* s.s., *An. gambiae* s.s.	CDC, PSC, backpack aspirator	Not evaluated	LLIN, IRS	All year with seasonal fluctuations	8–108 for *An. funestus*; 0–8 for *An. gambiae*
	Nchelenge District							
	Choma District	*Pf*	*An. arabiensis*	CDC, HLC, PSC, cattle-baited trap	Exophagic, exophilic	LLIN	Single rainy season	0
	Zimbabwe	*Pf*	*An. funestus* s.s., *An. gambiae* s.l.	CDC, PSC	Not evaluated	LLIN, IRS	Single rainy season	0–7 for *An. funestus*; N/A for *An. gambiae*
	Mutasa District							
East Africa	Uganda	*Pf*	*An. arabiensis*, *An. gambiae* s.s.	HLC, PSC/exit trap, CDC	Primarily endophagic, endophilic	ITN	Perennial, two annual rainy seasons	4
	Jinja District							
	Kanungu District	*Pf*	*An. gambiae* s.s.	HLC, PSC/exit trap, CDC	Primarily endophagic, endophilic	ITN	Perennial, two annual rainy seasons	27
	Tororo District	*Pf*	*An. gambiae* s.s.	HLC, PSC/exit trap, CDC	Primarily endophagic, endophilic	ITN	Perennial, two annual rainy seasons	125
Amazonia	Peru	*Pv*, *Pf*	*Anopheles darlingi*	HLC, CDC, Shannon trap	Exophagic, exophilic	LLIN, IRS, local bed nets	Seasonal, peaks w/rainy season (March–May)	0–86.7
	Loreto Department							
	Madre de Dios Department*	*Pv*	*Anopheles rangeli*, *Anopheles benarrochi* B	HLC, CDC, Shannon trap	Not evaluated	ITN	Perennial, peaks w/rainy season (December–February)	Too few mosquitoes to calculate
	Brazil	*Pv*	*An. darlingi*	Shannon trap	Primarily exophagic, exophilic	ITN, IRS	Seasonal; minor peaks during dry season (May–September)	N/A
	Granada, ∼25-year-old rural settlement, Acrelandia							
	Remansinho, ∼8-year-old settlement, Acrelandia	*Pv*	*An. darlingi*	Shannon trap	Exo/endophagic, primarily exophilic	ITN	Perennial†	N/A
Latin America	Colombia	*Pv*, *Pf*	*Anopheles nuneztovari*, *An. darlingi*	HLC	Exophagic, exophilic	LLIN	Perennial	Three localities for *An. nuneztovari*: 3.5, 3.2, 1.9
	Tierralta							
	Buenaventura	*Pv*, *Pf*	*An. nuneztovari*, *Anopheles pseudo punctipennis*, *Anopheles albimanus*	HLC	Exophagic, exophilic	LLIN	Perennial, modest peaks March–April, July–September	Too few mosquitoes to calculate
	Tumaco	*Pv*, *Pf*	*An. albimanus*, *Anopheles calderoni*	HLC	Exophagic, exophilic	LLIN, larvicide	Perennial, main peak March–April; minor peak July	2.85 for *An. calderoni*
South Asia	India	*Pv*, *Pf*	*Anopheles stephensi*, *Anopheles subpictus*	Mosquito magnet, CDC	Endophagic, exophilic; endophagic, endophilic	Larvicide (temephos), BTI, larvivorous fish (guppy)	All year, peaks during rainy season	2.35 overall for *An. stephensi* in multiple localities in Goa; 18.1 for Panaji (within Goa) alone
	Goa							
	Wardha	*Pv*, *Pf*	*Anopheles culicifacies*	Hand catch	Endophilic, endophagic	IRS	All year, peaks during rainy season	Unreported
	Ranchi	*Pv*, *Pf*	*An. culicifacies*, *Anopheles fluviatilis*	Hand catch	Endophilic, endophagic	LLIN, IRS	*An. culicifacies* all year, peaks post rainy season (southwest monsoons); *An. fluviatilis* peaks February–March	Unreported
	Assam	*Pv*, *Pf*, *Po*, *Pm*	*Anopheles baimaii*, *Anopheles minimus*	CDC	Exophilic, exo/endophagic; exo/endophagic	LLIN, ITN, IRS	All year, peaks during rainy season	Unreported
	Chennai	*Pv*, *Pf*	*An. stephensi*	IRC, PSC	Endo/exophilic (variable; mainly based on microenvironmental conditions) endophagic; exophagic during summer	Larvicide (temephos)	Perennial, mesoendemic, southwest and northeast monsoon (predominantly NE)	Unreported
	Raurkela	*Pv*, *Pf*, *Pm*	*An. fluviatilis*, *An. culicifacies*	IRC	*An. fluviatilis* classically endophilic and endophagic w/ evidence of switch toward exophily (cattle sheds) and more exophagy (zoophagy). *An. culicifacies* strongly zoophilic (rests, feeds in cattle sheds); any human feeding tends to be endophagic	IRS, LLIN	Perennial, meso-hyperendemic, peak in winter	7.3–127 seasonally dependent
	Nadiad	*Pv*, *Pf*	*An. culicifacies* A and C in rural areas	IRC, PSC	Endophilic, endophagic	Larvicide, biological control, IRS, LLIN, ITN	Seasonal, hypoendemic (unstable malaria)	0.05–0.21
Southeast Asia	China	*Pv*, *Pf*	*An. minimus*, *Anopheles maculatus*, *Anopheles sinensis*	CDC aspirator	Exo/endophilic; strongly zoophilic, exophagic	ITN, IRS	Perennial, one rainy season	0.10
	Yingjiang County, Yunnan Province							
	Myanmar	*Pv*, *Pf*	*An. minimus*, *An. maculatus*, *An. sinensis*	CDC	Exo/endophilic; strongly zoophilic, exophagic	LLIN, IRS	Perennial, one rainy season	0.53
	Laiza, Kachin State							
	Thailand	*Pv*, *Pf*	*An. minimus*, *An. maculatus*, *Anopheles annularis*	CDC aspirator	Exo/endophilic	LLIN, IRS	Perennial, one rainy season	0.25
	Tha Song Yang District, Tak Province							
Southwest Pacific	PNG	*Pv*, *Pf*, *Po*, *Pm*	*Anopheles punctulatus* complex	HLC, barrier screens	Exo/endophilic	LLIN	Perennial, one to two rainy seasons	10.1–27.8
	East Sepik Province							
	Madang Province	*Pv*, *Pf*, *Po*, *Pm*	*Anopheles farauti*	HLC, barrier screens	Exo/endophilic	LLIN	Perennial, peak in rainy season	40.8
	Solomon Islands	*Pv*, *Pf*, *Po*, *Pm*	*An. farauti*	HLC, barrier screens	Exophilic	LLIN	Perennial, peak in rainy season	3–44
	Central Province and Western Province							

ASP = battery powered aspirator of the Prokopack or Insectazooka type; CDC = Centers for Disease Control and Prevention; EIR = entomological inoculation rate; HLC = human landing catch; ICEMR = International Centers for Excellence in Malaria Research; IR = infection rate (in vector); IRS = indoor residual spray; IRC = indoor resting collections; LLIN = long-lasting insecticide-impregnated net; PNG = Papua New Guinea; PSC = pyrethroid spray catch; *Pf* = *Plasmodium falciparum*; *Pm* = *Plasmodium malariae*; *Po* = *Plasmodium ovale*; *Pv* = *Plasmodium vivax*; N/A = Not applicable.

Locality numbers in Column 2 correspond to numbers in Figure 1.

* Malaria cases in Madre de Dios Department have steadily declined since 2011. In 2013, there were 251 cases (MINSA, Peru, 2013).

† Malaria (*P. vivax*) is disappearing in Remansinho (2010–2013).^24^
